# The Races of Britain: a Contribution to the Anthropology of Western Europe

**Published:** 1886-03

**Authors:** 


*The Races of Britain: a Contribution to the Anthropology of Western Europe.* By JOHN BEDDOE, M.D., F.R.S., &c. Bristol: ARROWSMITH. London: TRÜBNER. 1885.

We have long held the opinion that what our predecessors dwelt so much on, and graphically divided into orders and sub-orders under the term of "temperament," has in modern pathology and prognosis been too slightingly regarded; the truth which underlay the views of older observers may be more accurately observed and defined by a study of anthropology. The correctness of this view may be easily conceived by studying the pathological tendencies of very distantly related races; for example, the Anglo-Saxon and the Malay, which we have observed in this city. In the different branches of the great Aryan family, these peculiarities are less distinctly marked, and almost imperceptibly shade off into one another, even in members of the same family, owing to the admixture of blood; still, the racial as well as the pathological differences of the phlegmatic German, of the dark Silurian, of the elastic springy Gael of Scotland and Ireland, and of the round-headed lively Frenchman of Central and Southern France, have a deep pathological import, which should be carefully studied, however minute, through crossing, the shades of differences may at first appear.

The limited space at our command precludes our doing justice to this extraordinary book, which has evidently been to the learned author a pure labour of love. We shall, therefore, only notice some points which prominently attract our attention.

This work is, so far as our knowledge extends, the first attempt to describe scientifically the races of the British Isles. The celebrated Paul Broca had done for France what no one previous to our author had ever attempted for these islands; and although we yield to no one in our admiration of the late Professor Broca, we consider that the task which he so ably accomplished was much easier than that of our author, as the racial elements which have entered into the composition of the French nation are fewer in number than those which comprise the population of these islands.

The author has advanced a most startling opinion; viz., that two strata of a pre-Aryan population underlay the subsequent Aryan additions of Celts, Romans, and Teutons; and by the minute details of the characteristics which he gives of the population at the present time, he shows that these pre-Aryan races are still represented among our people. There was a time when we would have combated such ethnological heterodoxy, but the logic of facts is indisputable. We have beyond question a considerable proportion of Allophyllian and Basque blood still amongst us, varying in proportion in different localities.

The author, on page 29, has given a short but masterly summary of the population of Britain before the landing of the Romans, which we commend for the careful study of everyone about to commence a course of reading of English history.

The author has given us a few well-marked typical portraits of some of the different racial elements among us at the present time, but only just enough to excite a desire for more. The portraits of the man of the Bronze age, still very common in England, of the Devonshire beauty, of the Aranmore Gael, of the woman west of Kerry, and of others, are excellent; but surely, in these days of dry plates and instantaneous photography, Dr. Beddoe could have given us a series of typical representations, comprising every form of distinctive human countenance that has entered into our national composition. The practised eye of the author could pick out excellent types of each at promiscuous assemblies in the country. A walk through local photographers' galleries would supply many good specimens. There are many English types known to us, which we hope to find represented in the next edition; for instance, we should like to see good typical portraits of the non-Aryan population, as found even in Central England in the present day. We would hail a good representation of the tall, dark, but fine-haired Englishman of the midland counties, who asserts his ancient race, although surrounded by a fair-haired Anglo-Danish population. This variety, we remember, attracted the special notice of the late Professor Rolleston. Good typical portraits of all the nationalities, placed in the order in which they entered, would be most instructive. This can be done, and the author is the one who can best do it. We hope he will not flinch from the task which his own accomplishments indicate to him.

Immense labour must have been expended by the author on the copious details of measurements and personal observations, and the excellent series of ethnological maps, British and Continental, which are given to illustrate the text. The author has taken nothing for granted; and he has severely restricted his imagination, and abstained from all speculation. His chief guides have been the eye, the calipers, the measure tape, and the scales. Possibly such a course was prudent in a first attempt to enlist interest in scientific anthropology; nevertheless, we have wished the author had generalised a little more, and grouped the numerous component nationalities a little more closely, and given us at least some idea of their respective moral and mental characteristics. We would fain believe that in this direction lies the true philosophy of history, and so of the British constitution. Possibly some future historian, working on the author's lines, will point out the hidden wheels of the machinery which have produced this great nation, and will prove that all the great events of our history issued from anthropological facts—that the Magna Charta, the Reformation, the incurable crookedness of the Stuarts, the Great Rebellion, the Revolution of 1688, the Reform Bill, the present Irish difficulty, as well as other matters of great moment, have all arisen from these; then, doubtless, this science of man demands our most serious attention.

Although this science has hitherto been chiefly looked upon as a mere subject for the amusement of the "dilettante," we trust this truly scientific work will attract the attention of earnest students to it, and that the time will soon arrive when the racial character of man will be deemed worthy of study, and that the race of man, as he appears in these islands, will in the future receive as much attention as has been profusely lavished on the short-horned ox and other families of the lower animals.

